# Female Genital Mutilation/Cutting Education for Midwives and Nurses as Informed by Women’s Experiences: Protocol for an Exploratory Sequential Mixed Methods Study

**DOI:** 10.2196/32911

**Published:** 2021-10-15

**Authors:** Monica Pilar Diaz, Mary Steen, Angela Brown, Julie-Anne Fleet, Jan Williams

**Affiliations:** 1 UniSA Clinical and Health Sciences Unit University of South Australia Adelaide Australia; 2 Women's and Children's Hospital Women's and Children's Health Network Adelaide Australia; 3 Refugee Health Service Central Adelaide Local Health Network Adelaide Australia

**Keywords:** education, midwives, nurses, female genital mutilation/cutting, maternity care, women’s health care, knowledge, attitude, practice

## Abstract

**Background:**

Female genital mutilation/cutting (FGM/C) is a complex and deeply rooted sociocultural custom that is innately entrenched in the lives of those who continue its practice despite the physical and psychological dangers it perpetrates. FGM/C is considered a significant independent risk factor for adverse maternal and fetal outcomes in pregnancy and childbirth. Several studies in high-income countries have explored the experiences and needs of women with FGM/C as well as the knowledge of the health professionals, particularly midwives and nurses, who care for them. However, to date, no studies have evaluated the implementation of education for health professionals in high-income countries to meet the specific needs of women with FGM/C.

**Objective:**

This study aims to explore the impact of an FGM/C education program for midwives and nurses as informed by the experiences of women with FGM/C accessing maternity, gynecological, and sexual health services in South Australia.

**Methods:**

This study will adopt a three-phase, exploratory sequential mixed methods design. Phase 1 will involve the *exploration* of women with FGM/C views and experiences accessing maternity and gynecological (including sexual health) services in South Australia. The findings from phase 1 will inform phase 2: the *development* of an educational program for midwives and nurses on the health and cultural needs of women with FGM/C. Phase 3 will involve the *evaluation* of the program by measuring midwives’ and nurses’ changes in knowledge, attitude, and practice immediately before and after the education as well as 4 months after completing the program. Phase 1 of this study has been approved by the Women’s and Children’s Health Network human research ethics committee (ID number 2021/HRE00156) and the University of South Australia human research ethics committee (ID number 204096).

**Results:**

Phase 1 will commence in August 2021, with the interpretation of findings being undertaken by November 2021. Phase 2 will be developed and facilitated by February 2022, and the final phase of this study will begin in March 2022. This study is expected to be completed by February 2023.

**Conclusions:**

The findings of this research will provide insight into the development and evaluation of education programs for midwives and nurses that includes collaboration with women from culturally and linguistically diverse backgrounds to address the specific cultural and health needs of communities.

**International Registered Report Identifier (IRRID):**

PRR1-10.2196/32911

## Introduction

### Background

The Australian Institute of Health and Welfare estimates that 53,000 girls and women living in Australia have undergone female genital mutilation/cutting (FGM/C) [1]. However, it is not clear how many girls and women are at risk of being illegally subjected to the practice as there are no formal guidelines, policies, or training for health professionals on the identification, reporting, and management of FGM/C cases in Australia [2]. In 2014, a report by Family Planning New South Wales found that the mandatory collection and reporting of FGM/C cases could only be achieved through adequate education and training of health professionals on FGM/C [3].

Girls and women with FGM/C can experience serious immediate and long-term health complications because of the practice [4]. Childbirth can be a particularly challenging time for women with FGM/C, as the physical and psychological complications of FGM/C may be exacerbated [5-10]. Furthermore, FGM/C is considered a significant independent risk factor for adverse maternal and fetal outcomes in pregnancy and childbirth [11]. Despite this, midwives and nurses often report that they are not equipped to effectively support women with FGM/C because of a lack of educational opportunities [12,13]. Some midwives and nurses report that they do not have the knowledge, clinical skills, cultural competence, confidence, and awareness of legal responsibilities to effectively support the needs of women with FGM/C, elements that are essential in the provision of woman-centered care [14].

Research on the effectiveness of FGM/C focuses on prevention programs; however, it has demonstrated that the key to success is through engagement and collaboration with women and affected communities [2,15,16]. With regard to exploring the experiences of women with FGM/C to assist in the design of education for midwives and nurses, there appears to be a gap in the literature. Therefore, this study protocol will engage and listen to women with FGM/C to assist in the development of an education program for health professionals (ie, midwives and nurses who work in maternity, sexual, and reproductive health services) and to improve midwives’ and nurses’ clinical skills, cultural competence, confidence, and awareness of legal responsibilities to effectively support the needs of women with FGM/C. This paper describes a study protocol for the development and evaluation of an educational module on FGM/C for midwives and nurses as informed by women’s experiences.

### Aims

This study aims to explore the views and experiences of women with FGM/C accessing maternity, gynecological, and sexual health services in South Australia to assist in the development of an educational program for midwives and nurses.

### Objectives

The objectives of this study are as follows:

- To design an FGM/C education module for midwives and nurses in collaboration with women who have experienced FGM/C to meet their cultural and health needs.
- To collaborate with key stakeholders to assist with the development of an FGM/C education and training program for midwives and nurses.
- To evaluate the impact of the FGM/C education and training program and assess the knowledge, attitudes, and practice (KAP) of midwives and nurses providing care to women with FGM/C.

### Research Questions

The research questions are as follows:

1. What are the views and experiences of women with FGM/C in South Australia?
2. What factors affect the experiences of women with FGM/C accessing maternity, gynecological, and sexual health services in South Australia?
3. What FGM/C education and training resources are currently available and accessible to midwives and nurses in Australia?
4. What impact does FGM/C education and training have on the knowledge, attitude, and clinical practice of midwives and nurses when supporting women with FGM/C?

## Methods

### Study Design

**Figure 1:**
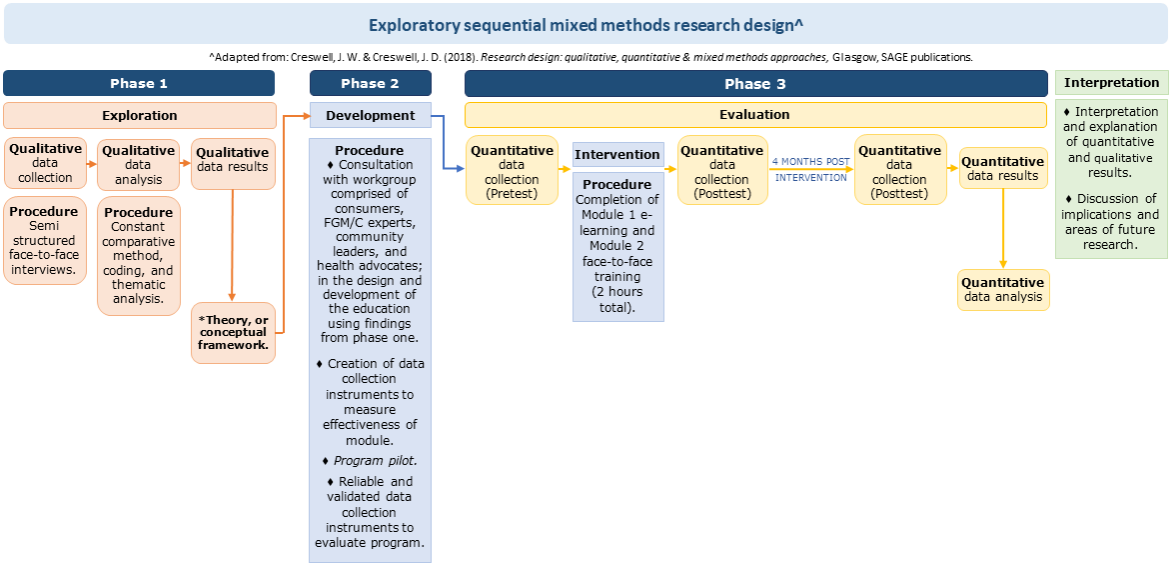
Exploratory sequential mixed methods research design [17]. FGM/C: female genital mutilation/cutting.

This proposed study will use a three-phase, exploratory, sequential mixed methods design (Figure 1). Phase 1 will be *exploration* of the views and experiences of women with FGM/C accessing maternity, gynecological, and sexual health services in South Australia. The findings from phase 1 will be used to inform phase 2: *development* facilitation of an FGM/C educational and training program for midwives and nurses. Phase 3 will involve the *evaluation* of the education and training program and measure the KAP of midwives and nurses before and after completing the course.

### Conceptual Framework

A mixed methods research design is a systematic way of *combining or integrating* qualitative and quantitative methods, data collection, and analysis to thoroughly examine a concept or issue that a single construct may not be able to capture completely [17,18]. This approach is pragmatic and enables researchers to be *open* to using a range of research methods, assumptions, and worldviews to attain a deeper understanding of the phenomenon under investigation [17]. Integrating qualitative and quantitative data in mixed methods allows each approach to complement each other, in turn, strengthening the findings and decreasing the weakness of using a single construct [17].

This proposed study will adopt an exploratory, sequential mixed methods design as the most suitable construct to answer the proposed research questions. Exploratory designs are data driven and enable researchers to *explore* the topic first where there is limited understanding of it or no prior conceptual frameworks [17].

### Setting

This research will be undertaken in metropolitan and regional South Australia. Participants will be sought from public, private, and community health sectors that provide maternity, gynecological, and sexual health care services.

### Sampling

A purposeful nonprobability sampling technique will be used to recruit participants for this study [19,20]. The aim of a purposive sample is to describe a group of participants with the same or similar characteristics (eg, women with FGM/C who have accessed health services in South Australia and registered midwives and nurses practicing in South Australia) [21].

### Participants

This study will recruit 2 different cohorts of participants for phases 1 and 3 of the study.

#### Phase 1 Participants

This includes women living in South Australia who self-identify as having FGM/C (types 1-4).

##### Inclusion Criteria

The criteria for inclusion are as follows: (1) women aged >18 years; (2) identify as having FGM/C (type 1-4); (3) reside in South Australia; and (4) have accessed maternity, gynecological, and sexual health services in South Australia in the past 5 years.

##### Exclusion Criteria

The criteria for exclusion are as follows: (1) women aged <18 years; (2) have not experienced FGM/C; (3) do not reside in South Australia; (4) have not accessed maternity, gynecological, and sexual health services in South Australia; and (5) are unable to provide informed consent. Please note that the ability of women to speak or understand the English language will not be a requirement in this research, as qualified interpreters will be used. Interpreters with experience in translating in health care settings from South Australian Health–approved interpreting services will be used in this study.

#### Phase 3 Participants

This includes midwives and nurses registered with the Australian Health Practitioner Regulation Agency.

##### Inclusion Criteria

The criteria for inclusion are as follows: (1) registered midwives and nurses, (2) working in clinical practice in South Australia, and (3) have cared for or will care for women with FGM/C.

##### Exclusion Criteria

The criteria for exclusion are as follows: (1) not a registered midwife or nurse, and (2) not working in clinical practice in South Australia.

### Recruitment

Recruitment for each phase will occur in 3 stages.

#### Stage One

In phase 1, participants will be recruited through the study’s website and the principal researcher. Advertisements and study website information will be placed on social media, and posters will be displayed at several clinical sites across South Australia. Information sessions will be provided to midwives and nurses in clinical settings to increase awareness of the study and how to advise potential participants to access the study’s website and contact information of the principal researcher. Women will be able to express their interest in the study by emailing or phoning the principal researcher directly or by registering their interest through the study’s website. The principal researcher will contact the women to discuss the study, answer any questions, and inform them of what is required to participate in the study.

#### Stage Two

In phase 2, participants will be women (from phase 1) and professionals who have expertise in supporting women who have experienced FGM/C, community leaders, and health advocates. The FGM/C experts, community leaders, and health advocates will be recruited through snowball sampling and via email, inviting them to be part of the collaboration group.

#### Stage Three

Phase 3 will be the recruitment of registered midwives and nurses currently practicing in South Australia, working in maternity, gynecology, and sexual health services. Participants will be invited to participate in phase 3, that is, the *evaluation* of an education and training program through advertisements on social media, the Australian College of Midwives, the Australian Nursing and Midwifery Federation, South Australia Health information sessions, and the University of South Australia (UniSA).

### Ethical Considerations

This project has ethical approval from the Women’s and Children’s Health Network human research ethics committee (ID number 2021/HRE00156) and the UniSA human research ethics committee (ID number 204096).

#### Consent

Participants in phase 1 will be provided with a participant information sheet (PIS) to read in plain English or translated to Arabic, Swahili, Somali, Tigrinya, Amharic, Indonesian, and Malay languages (common languages spoken by FGM/C-practicing communities [22]). The PIS will explain the details of the study, including the purpose of the research, participation requirements, confidentiality, data management, consent process, and how the research information collected will be used. Participants will have access to the PIS in a digital format on the study’s website or a hard copy in the form of a pamphlet.

For women who are unable to read English, an audio recording of the written information of the PIS and consent form will be available in their preferred language, and/or an interpreter will be available to read the forms to the women. At this stage, the women will be informed of the researchers’ intention to use their contact details to invite them to participate in a working group for phase 2 of the study.

Participants in phase 3 (midwives and nurses) will also be provided with a PIS specific to phase 3. The PIS will have detailed information regarding the purpose of the research, participation requirements, confidentiality, data management, consent process, and how the research information collected will be used. All participants (from phases 1 and 3) will be required to provide written consent before commencing the study, with a copy provided to them for their records.

#### Participant Safety and Withdrawal

Participation in the study will be voluntary. Participants can withdraw from the study at any time before the interviews take place and before the deidentification of transcript verbatim (phase 1) as well as before commencing the education program (phase 3). Any data collected up to the point of withdrawal will be included in the final data analysis. No additional data will be collected after withdrawal. Withdrawing from the study will not affect the woman’s access to health services.

### Cultural Considerations

Owing to the sensitive and emotive nature of FGM/C, there are several potential cultural issues that are anticipated by the researchers, and measures will be put in place to minimize the effect, as outlined in the sections below.

#### Reluctance to Speak About FGM/C

FGM/C is considered a taboo women’s issue and a topic that women are not willing to openly discuss. Feelings of shame and stigma associated with the criminalization of FGM/C in high-income countries may make women reluctant to disclose information surrounding the issue out of fear of discrimination or legal repercussions. Several studies have demonstrated that community support is the single most important aspect in establishing a strong rapport with women with FGM/C [16,23,24]. Therefore, to minimize these concerns, the researchers will liaise with community leaders, FGM/C clinical experts, and health advocates to gain their support and guidance to develop a framework on how to best support women participating in the study. Community leaders and FGM/C experts will also review the information and language used in the recruitment posters, postcards, PIS, and the study’s website.

#### Vulnerable Women, Domestic Violence, and Reliving Psychological Trauma

##### Overview

FGM/C is recognized as a violation of the human rights of girls and women globally [22]. The practice is a deeply embedded sociocultural tradition among practicing communities, posing many challenges to its eradication, as women can be both the victims and perpetrators of FGM/C [15]. Women with FGM/C are more likely to experience sexual intimate partner violence than women who have not had the procedure [25,26]. It is well documented that storytelling triggers several concerns for victims of abuse, particularly, reliving the psychological trauma and resurfacing of suppressed memories [7,27]. Anticipating these issues, the researchers will take steps to address these and minimize any distress for participants.

The following steps will be followed during recruitment

- Researchers will ensure that all potential participants will have a complete understanding of their rights to withdraw from the study without any consequences.
- Confidentiality will be maintained, and the benefits and risks of the study, privacy, and respect of women’s cultural values as detailed in the PIS and consent forms provided will be explained.
- In phase 1 recruitment stage, the researchers will ensure that women considering participating in the study understand that the research team is only interested in their experience in accessing health services in South Australia and not the events that led to FGM/C. However, if women wish to discuss their personal experiences, they will be supported during the interview to do so.
- Participants wishing to discuss any aspects of the study or concerns will be able to do so at any time by contacting the principal researcher directly (phone numbers and emails will be supplied).
- Full disclosure of researchers’ duty of care to ensure participants’ safety, including mandatory reporting under the *South Australian Criminal Law Consolidation Act 1935* and the *Children’s Protection Act 1993*, will be explained.
- The following steps will be followed while the study is being conducted: At the beginning of phases 1 and 3, the researchers will reiterate the information given to participants during recruitment and provide an opportunity for further questions.
- A safe, private environment (either at the UniSA City East campus or at a place where the woman feels most comfortable and of her choosing) will be used to undertake the interviews in phase 1.
- Participants who become distressed at any time of the study will be provided with an opportunity to take a break from the interview and only recommence if they wish to continue. A *participant support protocol pathway* (Multimedia Appendix 1) will be used to ensure that women are provided all the necessary support.
- Any intimate partner violence concerns identified during the study will be addressed by offering the participant support and referral information to specialized services (eg, Women’s Safety Services South Australia: migrant women support, Domestic Violence Gateway Service, National Sexual Assault Domestic Family Violence Counselling Service, Relationships Australia, South Australia Police and Specialized Family Violence Service, Survivors of Torture and Trauma Assistance and Rehabilitation Service, and Refugee Health Service).

##### After the Study

Participants will be provided with an opportunity to discuss any concerns at the end of their participation.

#### Confidentiality

The participants’ confidentiality will be maintained through a deidentified collection of data. In phase 1, interview transcripts will be given pseudonyms to separate the data but no identifiable characteristics will be collected to maintain confidentiality. All possible identifiable characteristics (names of people or places) in the transcripts will be omitted to prevent inadvertent deductive disclosure. In phase 3, the program evaluation questionnaires will be deidentified at the point of collection. Participants will be emailed a randomly assigned log-in code to access the education program. The code will be used by the participant for the duration of the study and will not be linked back to the participant. No identifiable characteristics will be used in the reporting of qualitative findings and quantitative results.

#### Data Management Plan

Participants’ confidentiality will be maintained through the deidentified collection of data. Interview recordings will be collected with a handheld voice recorder and erased after transcription. Questionnaires will be deidentified at the point of collection to prevent participants from being identified. The data management process will be organized according to the UniSA guidelines and using My Data Management Plan tool (a UniSA program).

Storage archiving of data will be stored on the web via software provided by the UniSA local server. Data files will be stored in at least 2 locations to reduce complete loss. Data will be stored on a USB drive and a PC (password protected) at UniSA. Data will be frequently backed up on the UniSA server. Data will remain confidential and limit access only to the research team. Hard copy data collection tools will be stored in a locked filing cabinet in a locked room at the UniSA City East campus. Measures will be taken to ensure the security of information from misuse, loss, or unauthorized access while stored during the research project. Research data and records will be maintained for 5 years after publication. This storage of data requirement complies with the ownership and retention of data policy, as outlined by the National Statement on Ethical Conduct in Human Research and UniSA data storage policy.

In terms of secure data destruction, the primary investigator will obtain written approval from the executive dean of the UniSA Clinical and Health Sciences Unit for secure destruction of research data, materials, and associated research records. This data material will be shredded and disposed of in confidential, secure document destruction bins provided at the university. All data stored electronically will be deleted through a process of repeated overwriting of the documents and deletion from the server, ensuring that the contents cannot be recovered.

### Procedure

#### Phase 1: Exploration

##### Interviews (Option 1)

Women will have the choice of attending an interview in person at UniSA City East campus or via telephone at a prearranged date and time.

Semistructured interviews will be used to explore the views and experiences of women with FGM/C who have accessed or are currently accessing maternity, gynecological, and/or sexual health services in South Australia for this phase. Semistructured questions will be asked by the researcher using examples identified from previous studies that have investigated the views and experiences of women with FGM/C [16,28]. The questions will be asked in a flexible manner and adapted, omitted, or elaborated on to correspond to the individual needs of each participant.

At the end of the interview, the principal researcher will provide participants with an opportunity to check and confirm responses and that these are a true representation of their views and experiences. If there is anything women would like to clarify or omit, they can do so. No further changes will be possible once data analysis or publication is conducted.

##### Data Saturation

Data saturation will be attained when no new themes are identified [29,30]. Data saturation will be guided by the Hennink et al [30] framework. These researchers reported that data saturation could be achieved with 10 interviews but recommended that researchers should conduct a further 3 interviews to ensure that no new themes arise.

##### Web-Based Survey (Option 2)

The investigators acknowledge that some women will not want to reveal that they have had FGM/C but may still want to share their views and experiences accessing maternity, gynecological, and sexual health services. Women who wish to remain anonymous will have the option to complete a web-based questionnaire on the study’s webpage. The web-based questions will be the same semistructured questions used in the face-to-face interviews. Women will be able to review their answers before submission.

##### Data Analysis

The interview data will be manually transcribed by the principal researcher using the manual transcription function on the NVivo 12 (QRS International) software within 24-48 hours. No data will be saved on the cloud function of the program. A second researcher will check the accuracy of the scripts transcribed verbatim.

Constant comparative method (CCM) will be used to analyze data and generate codes from themes [31,32]. CCM uses a systematic process for analyzing interviews by constantly comparing the data, from “incident to incident, concepts emerging from further incidents in new data, and concept to concept” [32] to generate a theory. This approach enables researchers to identify gaps that require more exploration in future interviews. CCM has been used extensively in grounded theory research and can also be used effectively within other qualitative research [31,32].

**Figure 2:**
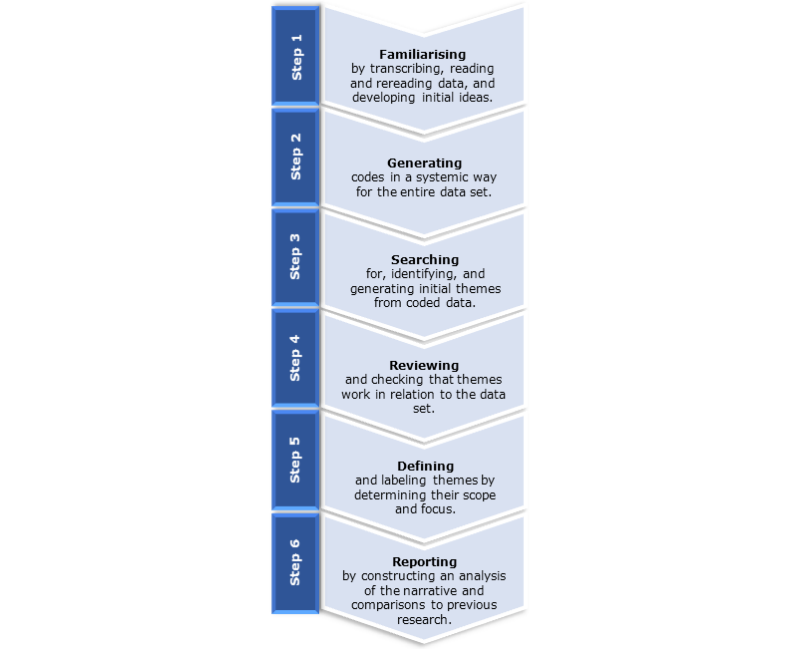
Braun and Clarke's [33] reflexive thematic analysis.

Themes will be generated using the 6 steps for reflexive thematic analysis by Braun and Clarke [33] (Figure 2). Any discrepancies or disagreements will be resolved through open discussions with the research team.

##### Gratuity

Women in the face-to-face and telephone interviews will be given an Aus $50 (US $37) gift card to reimburse their time and travel or parking costs.

#### Phase 2: Development

##### Overview

A workgroup collaborative will be established to develop the education and training program. The group will consist of women with FGM/C, health advocates, community leaders, and FGM/C clinical experts. The findings from phase 1 will inform the content of the program. The education program will be divided into 2 modules: module 1 (e-learning) and module 2 (face-to-face training).

##### Module 1: e-Learning

Module 1, an e-learning module, will cover the theoretical aspects of FGM/C, including, but not limited to the following:

1. Understanding FGM/C, including background and types of FGM/C
2. Understanding the law
3. Physical and psychosexual implications of FGM/C
4. Management of complications arising from FGM/C
5. Communicating with women living with FGM/C
6. Deinfibulation
7. Caring for women across the childbirth continuum
8. Additional resources

##### Module 2: Face-to-Face Training

Module 2 will be a 1-hour face-to-face interactive training session delivered at various clinical sites around South Australia. The session will provide participants with a summary of the information learned in module 1, a chance to discuss questions and any aspects of the course further, and an opportunity to consolidate their understanding of the theory. Phase 1 will inform the development of case studies that review the health needs and care of women with FGM/C in the antenatal, intrapartum, and postpartum periods based on the experiences of women. The training will involve group work and will be facilitated by the principal researcher. The content will include conducting sensitive history taking, effective documentation, use of interpreters, deinfibulation counseling, and physical examinations.

##### Data Collection Tools

KAP changes among midwives and nurses will be measured using 3 questionnaires: (1) a multiple-choice questionnaire to measure knowledge, (2) a 5-point-Likert scale survey to measure attitude, and (3) a survey to measure practice. The questionnaires will be developed based on previously validated questions identified in the literature that have evaluated FGM/C education for health professionals [34]. Knowledge will be measured via a multiple-choice questionnaire designed from the content of the program. Multiple-choice questionnaires are a convenient and effective assessment tool that has been extensively used in research to test knowledge [35-38].

Midwives’ and nurses’ attitudes toward FGM/C will be measured using 5-point Likert scales, adapted from 3 previous studies that have evaluated the implementation of education and training on FGM/C for health professionals [36,39,40]. Participants will be given a series of statements from which they will be required to select the extent to which they agree or disagree with each (ie, strongly agree, agree, neutral, disagree, and strongly disagree).

Information on the experience (practice) of midwives and nurses caring and supporting women with FGM/C will be collected through a survey that covers midwives’ and nurses’ experiences with FGM/C history taking, physical examinations, reinfibulation, mandatory reporting, and care plans.

Participants’ demographics, such as age, qualifications, years of clinical practice, and area of expertise, will be collected only once via a survey that will be completed on the web before commencing module 1.

##### Content Validity

Content validity of the FGM/C education program and data collection tools will be conducted by an expert panel of clinicians, FGM/C experts, and nursing and midwifery academics. Feedback will be sought on the content, wording, and structure of the education program and questionnaires and instructions to participants. Any disagreements among the members of the expert panel will be addressed through discussion and changes to the content until a consensus is reached [41].

##### Test-Retest Reliability

Test-retest reliability will be used to measure the reliability of the validated questionnaire [42]. Midwife or nurse volunteers will be invited to complete the questionnaires at the same time and then 2 weeks later. Correlations between the scores at both time points will be measured, and the instruments will be determined to be reliable or not. Reliability refers to the consistency of the tool that measures the same trait in the same person and situation at different points in time [42]. Instruments that are reliable guarantee the accuracy and replicability of the results in future studies by reducing random error and bias [43].

##### Pilot of Module 1

The education program (including questionnaires) will be piloted with a sample of 10 midwives and nurses who will not be involved in the study. These participants will be asked to complete an evaluation form to inform the researchers if any changes need to be made. The information that will be sought includes the relevance of the module to practice, navigation of the module, ease of access to the content, relevance of questionnaires, and time it took to complete.

##### Sample Size

Owing to the lack of previous data on the effect of FGM/C education on practicing midwives’ and nurses’ knowledge and attitudes, sample size calculation was conducted to detect a standardized medium effect (*d*=0.5). Reflective of the pre- and posttest design, a paired sample size calculation was conducted using the SAS/STAT version 14.2 software at a significance level of 0.05 and power of 0.9. Using a two-sided test and allowing for 20% loss to follow-up, a sample size of 55 nurses or midwives will be required to complete phase 3.

#### Phase 3: Evaluation

Midwives or nurses who consent to participate in the study will be provided a log-in to the web-based module using a personal code and password that will be emailed to them before the commencement of the study. Instructions on how to navigate the module and complete the assessments will be provided at the start of the program. Participants will be asked to complete a demographic questionnaire to gather information about their characteristics before commencing the module. Participants will then complete the 3 KAP questionnaires (pretest). Each question must be answered before the next one is provided to reduce nonresponse bias [44]. Access to the content of the module will only occur once the questionnaires are completed and submitted. The results will not be provided to avoid participants remembering their answers for the follow-up questionnaire posttest. At the completion of module 1, participants will be prompted to repeat the knowledge and attitude questionnaires only (posttest). Participants will be encouraged to complete the module and questionnaires on the same day to avoid any changes to the domains being tested, which could be caused by other types of learning outside of the intervention.

After 4 weeks of completion of module 1, all participants will receive an email with instructions for enrolling in module 2 (in-person training). Various dates, times, and venues will be provided during the consent stage to maximize attendance of module 2. Participants who attend the sessions will be asked to complete all 3 KAP questionnaires one more time at the beginning of the training day. The questionnaires will be paper based because of convenience, and it is less expensive than providing computer access to all the participants. This should reduce the attrition commonly observed in longitudinal studies [45,46]. At the end of the workshop, participants will be provided with a certificate of completion and 2 hours of continuing professional development points.

Phase 3 data will be analyzed using the SAS/STAT 14.2. Descriptive statistical analysis will be used to describe participants’ baseline characteristics. Continuous variables will be described using means and SDs. Categorical variables will be presented as frequencies and proportions. A paired sample two-tailed *t* test will be used to analyze the differences in knowledge and attitudes before and after the workshop, respectively. McNemar test compares the binomial proportions in a paired sample. It will be used in the study to test the changes in practice before and after the workshop.

## Results

The sequential exploratory design nature of this study will help researchers develop an educational module for midwives and nurses that is inclusive of the cultural and health needs of women with FGM/C. The data-driven nature of the exploratory mixed methods design allows the exploration of the topic where there is currently limited evidence—in this case, the experiences and health needs of women with FGM/C accessing maternity, gynecological, and sexual health services in South Australia.

This study will begin with an exploration of the topic in phase 1 to hear from women with FGM/C. This will be undertaken through face-to-face interviews and/or web-based questionnaires (open-ended questions) that will gather qualitative data. The findings will be analyzed and used to inform the development of an FGM/C bespoke education program for midwives and nurses as a way of *connecting* the data in phase 2 [17]. The education program will then be evaluated using pre- and posttest questionnaires to produce quantitative data in phase 3. This process is known as sequential, where one phase of the mixed methods informs or *builds* on the next [18].

## Discussion

### Importance of This Research

Notwithstanding worldwide campaigns to put an end to FGM/C, its prevalence continues because of a combination of cultural, religious, and social factors [22]. The World Health Organization estimates that 200 million girls and women have had FGM/C, and another 3 million are at risk every year [22]. Girls and women with FGM/C can suffer from serious short-and long-term complications, including physical, sexual, and psychological issues because of this practice [47]. FGM/C is considered an independent risk factor for adverse maternal and fetal outcomes in pregnancy and childbirth, resulting in a substantial financial impact on health services [48]. Women with types 1 and 2 of FGM/C are 3 and 5 times, respectively, more likely to sustain severe perineal trauma (third and fourth degree tears) than women who have not had the procedure [47].

In 2013, the Australian government made a commitment to end FGM/C by funding community awareness and education, reviewing Australia’s legal framework, and encouraging research and data collection to obtain the necessary evidence to support women and girls with FGM/C in Australia [49]. However, the implementation of these strategies has been slow to reach communities, and the practice continues. A total of 4 people have recently been convicted in Australia, in 2 separate cases, for performing FGM/C on a child and taking another overseas for the procedure [50,51]. These convictions highlight the need for more education and research in Australia and the mandatory reporting of FGM/C cases.

The most recent National Strategic Directions for Australian Maternity Services aims to ensure that maternity services in Australia are *“*equitable, safe, woman-centred, informed, and evidence-based” [52]. As such, women of culturally and linguistically diverse backgrounds need to have access to services that are responsive to their cultural needs, including access to interpreters, culturally trained health professionals, and bilingual and bicultural health advocates. The report informs the need to have routine antenatal identification of women with FGM/C and associated risk factors. This research will contribute to improving health services for women with FGM/C and provide evidence to inform health policies.

Phase 1 of this study is expected to be completed by November 2021. Phase 2 will commence from December 2021, with the aim of undertaking phase 3 in February 2022.

### Conclusions

As far as the authors are aware, there are no other studies that have used the views and experiences of women with FGM/C, or demonstrated a collaboration with relevant stakeholders, to develop an education program to educate midwives and nurses on the cultural and health needs of women from culturally and linguistically diverse backgrounds.

The findings and results from this exploratory mixed methods study will be collated, and meta-inferences will be developed and discussed. This research will be disseminated via publications and conference papers. The project outcomes will inform the provision of woman-centered health care education for midwives and nurses in South Australia.

## Appendix

Multimedia Appendix 1 Support protocol for face-to-face interviews.

Multimedia Appendix 2 Peer-review report (reviewer 1) by University of South Australia.

Multimedia Appendix 3 Peer-review report (reviewer 2) by University of South Australia.

## Abbreviations

CCM: constant comparative method
FGM/C: female genital mutilation/cutting
KAP: knowledge, attitude, and practice
PIS: participant information sheet
UniSA: University of South Australia
