# Supplementary material for: Female Genital Mutilation/Cutting Education for Midwives and Nurses as Informed by Women’s Experiences: Protocol for an Exploratory Sequential Mixed Methods Study
Source: JMIR Res Protoc. 2021 Oct 15;10(10):e32911. doi: 10.2196/32911 (PMC8556634; doi:10.2196/32911)
Supplement: Multimedia Appendix 1 [file resprot_v10i10e32911_app1.pdf]

SUPPORT PROTOCOL FOR FACE-TO-FACE INTERVIEWS WITH MIGRANT AND REFUGEE WOMEN[1]

SIGNS OF DISTRESS

At any stage of the interview if a woman:

- States that she is feeling anxious, upset, experiencing distressing flashbacks, or,
- Shows physical signs (e.g., crying, shaking, withdrawing, etc.) that the interview is stressful.

STOP

The interview immediately & **ASSESS**.

ASSESS

Ask:

- Are you okay?
- Would you like to end the interview now? Take a break? Or continue with the interview?

If the woman is no longer showing signs of distress or states that she would like to continue with the interview, follow the **PROCEED pathway.*

***If there are any indications that the woman is not coping or does not wish to continue with the interview follow the **END** pathway.*

*PROCEED

If the woman is no longer demonstrating signs of distress, or states to be okay, and consents; the interview may recommence (continue to monitor for any further signs of distress for the duration of the interview).

**END

If the woman is unable to continue the interview, provide her:

- With a safe/ quite space away from interview area to take time to recover.
- An opportunity to discuss her concerns.
- With the opportunity to call a family member or close friend (if one is not already present) for support or with consent, the principal researcher will call **Refugee Health Service** on behalf of the woman for phone counselling support with a community health advocate or nurse.

Once she feels safe to go home ensure that:

- She has a safe way to get home,
- She has a mental health support card (with useful numbers),
- She has the principal researcher's contact details if she has any further concerns, and
- Encourage her to contact GP or mental health provider for further support.

N.B: If the woman reveals information regarding a problematic episode of care at a healthcare facility or institution the researcher will provide the woman with the SA Health's "your feedback is important" consumer information sheet which explains how to lodge a complaint or provide service feedback.

FOLLOW UP

If the woman provides consent:

- Follow up with a courtesy phone call within 48hrs post interview to check distress status.
- Any further concerns must be directed to **Lifeline** (13 11 14) or **Refugee Health Service** (8237 3900) for support.

1. Modified from Draucker CB, Martsolf DS & Poole C 2009, 'Developing distress protocols for research on sensitive topics', *Archives of Psychiatric Nursing,* vol. 23, no. 5, pp. 343-350, & Haigh C & Witham G 2015, *'Distress protocol for qualitative data collection'*, Manchester Metropolitan University, Manchester.
