# Supplementary material for: Female Genital Mutilation/Cutting Education for Midwives and Nurses as Informed by Women’s Experiences: Protocol for an Exploratory Sequential Mixed Methods Study
Source: JMIR Res Protoc. 2021 Oct 15;10(10):e32911. doi: 10.2196/32911 (PMC8556634; doi:10.2196/32911)
Supplement: Multimedia Appendix 2 [file resprot_v10i10e32911_app2.pdf]

Confirmation of Candidature

Panel Assessment Form

**Name of Candidate**	**Monica Pilar Diaz**
**Name of Panel Member**	**Annette Briley**

My opinion of the students' proposal is summarised below *[Tick where appropriate]*

**Topic**	**Satisfactory**	**Unsatisfactory**
The topic contributes new knowledge to the subject area	√	
The topic has sufficient scope for doctoral research	√	
The proposed research has a sound policy, philosophical, scientific or theoretical base	√	
The proposed application of the research to issues in health and health care is discussed	√	
To my knowledge, the material in this proposed research has not been published before	√	

**Literature Review**	**Satisfactory**	**Unsatisfactory**
Succinct summary of the relevant literature was provided in the proposal	√	
The literature cited by the student is based chiefly on primary sources	√	
The sources of evidence on which the proposed research is based are clearly stated	√	
The interpretations and conclusions are justified by the evidence presented	√	

**Proposal**	**Satisfactory**	**Unsatisfactory**
The title of the research accurately reflects the content of the proposal	√<br>Could be tweaked to something like: The effectiveness of an educational program for midwives and nurses, about FGM and informed by women's experiences	
Clear and logical description of proposed research is presented	√	

The writing style is grammatically correct and references are cited appropriately and correspond accurately to the conventions used by the School		
A 300 word abstract is included in the proposal that accurately reflects the proposed research	√	
The research proposal does not exceed 20 pages	√	
Sufficient detail of proposed research is provided to allow evaluation	√	

**Research Method**	**Satisfactory**	**Unsatisfactory**
There is a clear statement of the purpose, aim, question or hypothesis of the research	√	
All key concepts are clearly defined (as appropriate)	√	
Research participants are clearly described. *The description covers (as appropriate) the inclusion criteria, identification and recruitment of participants, and justification of sample size*	√	
Data collection process is clearly described. *The description covers (as appropriate) what data will be collected, how the data will be collected and the processes that will be used to ensure accuracy of data. Proposal includes any data collection tools, scales or instruments that will be used*	√	
The proposed data analysis is clearly described, justified and appropriate	√	
The ethical aspects of the research are addressed (as appropriate)	√	
The ethical approval and any other permissions that are required to conduct research are described	√	
Any resource implications of the proposed research are adequately addressed	√	
Timeframe of the proposed research is described and is appropriate	√	
Trial Table of Contents is appropriate	√	

Are there any cost implications for this proposed study? Yes √☐ No ☐

If yes, have these costs been addressed in the proposal? Yes √☐ No ☐

Reviewer feedback to candidate

Please see comments on the research plan. This is important work. Some of my comments may be irrelevant here in Australia as I am unfamiliar with professional obligations re reporting. I am also unsure why going back to women who have accessed health care in SA since 1997 would be relevant. I would have thought a prospective or at least contemporaneous cohort might be more generaliseable moving forward.

Please include additional typed information, comments and critique of the proposal that can be sent to the candidate and supervisors

Reviewer signature: __________ __________________________ Date: 10/09/20
____
