# Supplementary material for: Female Genital Mutilation/Cutting Education for Midwives and Nurses as Informed by Women’s Experiences: Protocol for an Exploratory Sequential Mixed Methods Study
Source: JMIR Res Protoc. 2021 Oct 15;10(10):e32911. doi: 10.2196/32911 (PMC8556634; doi:10.2196/32911)
Supplement: Multimedia Appendix 3 [file resprot_v10i10e32911_app3.pdf]

University of South Australia

Confirmation of Candidature

Panel Assessment Form

**Name of Candidate**	**Monica Diaz**
**Name of Panel Member**	**Lois McKellar**

My opinion of the students' proposal is summarised below *[Tick where appropriate]*

**Topic**	**Satisfactory**	**Unsatisfactory**
The topic contributes new knowledge to the subject area	This is unclear at this stage and would be helpful to clearly outline the new knowledge this will contribute	
The topic has sufficient scope for doctoral research	Yes	
The proposed research has a sound policy, philosophical, scientific or theoretical base	Yes	
The proposed application of the research to issues in health and health care is discussed	Yes – very clear	
To my knowledge, the material in this proposed research has not been published before	Yes	

**Literature Review**	**Satisfactory**	**Unsatisfactory**
Succinct summary of the relevant literature was provided in the proposal	Excellent review of the literature having extensive sources and providing a clear and thorough background and context to the issue	
The literature cited by the student is based chiefly on primary sources	Yes	
The sources of evidence on which the proposed research is based are clearly stated	Yes	
The interpretations and conclusions are justified by the evidence presented	Yes	

**Proposal**	**Satisfactory**	**Unsatisfactory**
The title of the research accurately reflects the content of the proposal	Mostly, but as suggested below there needs to be more correlation to ensure that the education outcome is informed by the women	

Clear and logical description of proposed research is presented	Yes	
The writing style is grammatically correct and references are cited appropriately and correspond accurately to the conventions used by the School	Very well written	
A 300 word abstract is included in the proposal that accurately reflects the proposed research	Yes	
The research proposal does not exceed 20 pages	The margins are very narrow and the line spacing is 1 point this is not usually recommended for assessment reading – expectation is that this would have been 1.5 and this would have well exceeded proposal of 20 pages –but a very minor point as I am not sure you could have written this in less	
Sufficient detail of proposed research is provided to allow evaluation	Yes	

**Research Method**	**Satisfactory**	**Unsatisfactory**
There is a clear statement of the purpose, aim, question or hypothesis of the research	Yes	
All key concepts are clearly defined (as appropriate)	Yes	
Research participants are clearly described. *The description covers (as appropriate) the inclusion criteria, identification and recruitment of participants, and justification of sample size*	Yes	
Data collection process is clearly described. *The description covers (as appropriate) what data will be collected, how the data will be collected and the processes that will be used to ensure accuracy of data. Proposal includes any data collection tools, scales or instruments that will be used*	Yes	
The proposed data analysis is clearly described, justified and appropriate	Yes	
The ethical aspects of the research are addressed (as appropriate)	Yes	
The ethical approval and any other permissions that are required to conduct research are described	Yes	
Any resource implications of the proposed research are adequately addressed	Yes – though quite costly	
Timeframe of the proposed research is described and is appropriate	Yes	
Trial Table of Contents is appropriate	Yes	

Are there any cost implications for this proposed study? Yes ☒ No ☐

If yes, have these costs been addressed in the proposal? Yes ☒ No ☐

Reviewer feedback to candidate

Monica you have presented a well written and clear research proposal. The background literature is substantial and sets the scene for this research. You have provided a sound description of your research methodology and I must congratulate you on your excellent diagrams throughout. This is a research area that clearly needs further exploration and I recognise that it is a challenging area of research, I applaud you for the courage to take this on.

I acknowledge that I do not have the knowledge and understanding around this topic as you do, therefore I provide feedback from a researcher perspective. I have some concerns with the approach adopted to undertake research on FGM/C given the complex nature of the issue and the entrenched beliefs surrounding this from different perspectives. I would encourage you to think about incorporating a more cyclic approach rather than the linear design described in this proposal. This may require some more rethinking and reworking, but the end product will be significantly enhanced. At this stage, while it will contribute to research knowledge, this research appears to lack a novel aspect that sets it apart from research already undertaken. I have noted some further considerations below:

Being such a complex area and fraught with cultural challenges I wonder if undertaking other forms of qualitative data collection to supplement the interviews would be valuable – ie asking women to keep a journal for two weeks reflecting on their experiences in the past/currently with the health care system this could be provided completely anonymously and distributed by a community representative. You could set up a social media chat site, where the conversation could be interactional, or where women could add to other's thoughts, perpetuating exploration. I also think it is worth including midwives/nurses in stage one ie what are the midwives understanding of FGM and how women experience their care? It would be good to explore their knowledge, attitude and practice as part of the qualitative investigation, particularly as you have made this geographically specific ie SA –You could then consider how the experiences of these women and midwives sits within the broader international data.

At this stage I do not see a clear link between phase one and phase two. Phase two appears to be very predetermined. Interestingly, there is only one question in the interview guide on educational needs of the health professional - what if the qualitative data leads you somewhere else – ie professional bias, cultural training, inclusion in decision making. You need to clearly demonstrate what your research adds – what do these women want midwives/nurses to know beyond the facts that are available in the already established education resources ie WHO handbook? I think you have potential to really capture something novel – that is to develop a resource that really grasp the needs of the women and can relate this to the needs of the professional in being able to provide appropriate care. Maybe this would be a resource focussed on helping midwives/nurses unpack their own bias in relation to FGM/C, or how can they be present in a non-judgemental way, how do they process the paradox of cultural norm with abuse/criminal practice? At this

point I just don't see where the qualitative data is really influencing your outcome? This is an important aspect of the mixed method approach.

I encourage you to rethink some aspects of the research design, the educational outcome should be influenced by and acceptable to the women, for midwives/nurse. There should be women involved in developing the outcome and taking the learnings from the qualitative research, interpreting this together. I also think that it would be helpful to have midwives/nurses engaged in this aspect of creating a resource to ensure acceptability for the end user. I would encourage you to look at participatory research, cocreating, codesign etc – or simply create an advisory group from the women you interview and nurse/midwives and maybe others ie experts in FGM/C so that the outcome is being developed with them. I recognise that adding these elements would expand your research considerably but you could do a simpler evaluation at this stage (pilot), ready to implement and evaluate the impact on midwives' knowledge and attitudes, as well as the change to practice and the experience of women, as a postdoc.

I wish you all the very best on this exciting research.

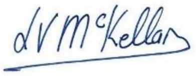

Reviewer signature: Date: 25-9-20
